# Selenium Kinetics in Humans Change Following 2 Years of Supplementation With Selenomethionine

**DOI:** 10.3389/fendo.2021.621687

**Published:** 2021-03-29

**Authors:** Blossom H. Patterson, Gerald F. Combs, Philip R. Taylor, Kristine Y. Patterson, James E. Moler, Meryl E. Wastney

**Affiliations:** ^1^ Biometry Research Group, Division of Cancer Prevention (DCP), National Cancer Institute, Bethesda, MD, United States; ^2^ Division of Nutritional Sciences, Cornell University, Ithaca, NY, United States; ^3^ Division of Cancer Epidemiology and Genetics, National Cancer Institute, Bethesda, MD, United States; ^4^ Beltsville Human Nutrition Research Center, United States Department of Agriculture-Agricultural Research Service (USDA-ARS), Beltsville, MD, United States; ^5^ Information Management Services, Inc., Rockville, MD, United States; ^6^ Metabolic Modeling Services, West Lafayette, IN, United States

**Keywords:** selenium, metabolism, trace elements, selenite, selenomethionine, kinetics

## Abstract

**Background:**

Selenium (Se) is a nutritionally essential trace element and health may be improved by increased Se intake. Previous kinetic studies have shown differences in metabolism of organic *vs.* inorganic forms of Se [e.g., higher absorption of selenomethionine (SeMet) than selenite (Sel), and more recycling of Se from SeMet than Sel]. However, the effects on Se metabolism after prolonged Se supplementation are not known.

**Objective:**

To determine how the metabolism and transport of Se changes in the whole-body in response to Se-supplementation by measuring Se kinetics before and after 2 years of Se supplementation with SeMet.

**Methods:**

We compared Se kinetics in humans [n = 31, aged 40 ± 3 y (mean ± SEM)] studied twice after oral tracer administration; initially (PK1), then after supplementation for 2 y with 200 µg/d of Se as selenomethionine (SeMet) (PK2). On each occasion, we administered two stable isotope tracers of Se orally: SeMet, the predominant food form, and selenite (Na_2_^76^SeO_3,_ or Sel), an inorganic form. Plasma and RBC were sampled for 4 mo; urine and feces were collected for the initial 12 d of each period. Samples were analyzed for tracers and total Se by isotope dilution GC-MS. Data were analyzed using a compartmental model, we published previously, to estimate fractional transfer between pools and pool masses in PK2.

**Results:**

We report that fractional absorption of SeMet or Sel do not change with SeMet supplementation and the amount of Se absorbed increased. The amount of Se excreted in urine increases but does not account for all the Se absorbed. As a result, there is a net incorporation of SeMet into various body pools. Nine of the 11 plasma pools doubled in PK2; two did not change. Differences in metabolism were observed for SeMet and Sel; RBC uptake increased 247% for SeMet, urinary excretion increased from two plasma pools for Sel and from two different pools for SeMet, and recycling to liver/tissues increased from one plasma pool for Sel and from two others for SeMet. One plasma pool increased more in males than females in PK2.

**Conclusions:**

Of 11 Se pools identified kinetically in human plasma, two did not increase in size after SeMet supplementation. These pools may be regulated and important during low Se intake.

## Introduction

Selenium is an essential nutrient for health, and there are indications that higher Se intakes may prevent certain diseases (1). There are 25 proteins that contain Se, as the amino acid selenocysteine (Sec), distributed in tissues throughout the body (2). These selenoproteins function in many systems including the endocrine, nervous, and immune systems (2). Selenium may have a role in some forms of cancer, cardiovascular disease, and cognitive decline (2) and relationships have been proposed between blood Se concentration and some health effects (1). However, assessing Se status is challenging because the element is typically consumed in several forms that are metabolized differently (3). Specifically, Se exists in nature in organic forms, such as selenomethionine (SeMet). SeMet can be converted to the functional form of Se, Sec, but SeMet can also be incorporated into proteins non-functionally in place of the amino acid methionine (3). An inorganic form of Se, selenite (Sel), often used as a supplement (3), has lower absorption than SeMet (4–6) and is only incorporated into functional selenoproteins (3). As total Se concentration in plasma and tissues includes both functional and non-functional selenoproteins, speciation is necessary for determining functional Se levels in tissues but many selenoproteins are low abundance making their detection challenging (7).

Selenium supplementation has been used to improve Se status in populations where soil Se is low [for a review see (8)]. People in the US are considered to have adequate Se intakes (i.e., >55 µg/d) (to convert µg/d to µmol/d, multiply by 0.0127) (9). Even so, 51% percent of the US population take nutritional supplements, many of which may contain Se (10). Use of Se-containing supplements has likely been increased as a result of findings reported from the Nutritional Prevention of Cancer (NPC) Trial in the US that supplementing skin cancer patients with Se significantly decreased the incidence of several cancers (lung, prostate, and colon) (11).

In part, because of the positive results from the NPC Trial, the Selenium and Vitamin E Cancer Prevention Trial (SELECT) was undertaken in some 35,000 healthy US men (12). The trial was stopped after 5 y because no effect was detected on prostate cancer incidence (13). However, both the choice of participants and form of Se used (SeMet) in SELECT have been questioned. It has been pointed out that the negative results in the SELECT cohort, which had relatively high Se status (average plasma Se >130 ng/ml), were consistent with the NPC results (11), which found no protective effects for subjects with relatively high Se status, e.g., >120 ng/ml (14). In addition, a non-significant association with risk for type-2 diabetes was noted in SELECT subjects supplemented with SeMet (13), although with additional follow up found that association to be further attenuated and remained non-significant (15). The NPC trial showed a significant positive association with self-reported diabetes based on 97 cases in all trial participants (hazard ratio = 1.55; 95% confidence interval 1.03–2.33) that was strongest in participants whose baseline plasma Se levels were highest (hazard ratio = 2.70 [1.30–5.61] among 37 cases with plasma Se >121.6 ng/ml) (16).

In order to target persons who might benefit most from Se supplementation, a better understanding of Se metabolism is needed (17). One approach to investigating metabolism *in vivo* is through the use of kinetic studies with tracers. Kinetic studies have been performed in humans after supplementation with various forms of Se for periods of up to a year (18–21); however, these kinetic studies were conducted for less than 3 wk. The objective of the current study was to compare the kinetics of two Se-forms (selenite [Sel] and SeMet) in healthy participants who were studied for a prolonged period (4 mo) before [published previously (6)] and after long-term (2 y) supplementation with SeMet, the dominant form of Se in foods (22). Kinetics were followed using stable Se isotopes and data were analyzed by compartmental modeling (6). The goal was to gain insight into how the metabolism and transport of Se changes in the whole-body long-term with increased Se intake, by identifying pools and pathways that responded to supplementation.

## Materials and Methods

Aspects of the study are presented in greater detail in Wastney et al. (6). They described model development and analysis of the first part of the study (see below in *Study Design*), however, the materials and methods were the same for the entire study.

### Participants

Participant recruitment and eligibility have been described elsewhere (23). Briefly, participants were non-smoking, healthy men (n = 16) and women (n = 15) aged 20–60 y, within 20% of their ideal weight, consuming regular diets, not taking Se supplements >25 µg/d, who ranked high on a Health Consciousness Scale (24), and whose plasma Se levels were 80–160 ng/ml. For reasons given in Wastney et al. (6), the final analyzable sample consisted of 7 males and 13 females. The study protocol was approved by the NCI Special Studies Institutional Review Board, the Cornell University Committee on Human Subjects, the Johns Hopkins University Human Subjects Committee (for the BHNRC).

### Study Design

The study consisted of three periods: an initial pharmacokinetics study of 4 mo duration (PK1); a 2 y period of Se-supplementation with 200 µg of Se/d as SeMet; a second, 4 mo pharmacokinetic study (PK2), identical in design to PK1, during which participants remained on supplement. The study lasted for a total of 32 mo. The objective of the design was to compare parameter values for the same participants before (PK1) and following long-term supplementation (PK2). At the beginning of each pharmacokinetic study, fasted participants were given two 300 µg tracer doses of two stable isotope tracers, 150 µg of Se as SeMet, and 150 µg of Se as Sel, orally, in water, on days 0 and 10. Based on results of previous studies (5, 25), two doses of tracer were given to ensure detection for the entire 4 mo periods of observation, PK1 and PK2. The stable isotopes were ^74^Se as L-selenomethionine (^74^SeMet, Amersham Laboratories, Chicago, USA), and ^76^Se as sodium selenite (Na_2_^76^SeO_3_, Oak Ridge National Laboratory, Oak Ridge, TN, USA) prepared as described in (6).

### Supplement

The supplement was produced under an Investigational New Drug Application (IND) obtained by the National Cancer Institute. The capsules were manufactured by University Pharmaceuticals of Maryland, Inc. from a specialty blend of L-SeMet and lactose called l-SeMet 5000 provided by Sabinsa Corp. N.J. Two lots of L-SeMet capsules were tested for appearance, assayed for content, content uniformity, variation in weight and dissolution.

### Sampling

Multiple blood, urine, and fecal samples were collected during both PK1 and PK2 (6). Specimens were sampled for 4 mo after administration of the initial doses of tracers. Blood (10 ml) was drawn immediately before, then at increasing intervals after administration of the tracer doses starting at 30 min. Complete urine and fecal collections were obtained for days 2–12 after the initial dosing.

### Chemical Analysis

Urine, fecal, RBC, and plasma samples were analyzed for both Se tracers and total Se. Following digestion and chelation, the ^74^Se, ^76^Se, and total Se contents of the samples were determined by triple isotope dilution gas chromatography-mass spectrometry using enriched ^82^Se as the internal standard (26). We measured these tracers but did not determine their chemical forms in our samples. While ^74^Se was given as SeMet, the measured species was not SeMet, *per se*, but was instead derived from SeMet. Thus, we have noted the measured species in italics, i.e., *SeMet* refers to a Se-containing compound originating as SeMet. Similarly, *Sel*, given as ^76^Se, refers to a species originating as Sel. Sel includes other Se forms that are metabolized similarly to Sel (e.g., SeCys). We do not make this distinction when reporting on the literature.

Concentrations (ng/g) of total Se, ^74^Se, and ^76^Se in plasma were converted to total masses by correcting for plasma specific gravity (assumed to be 1.026) and plasma volume (assumed to be 4% of body weight) (6). The same measurements in RBC were converted by correcting for RBC specific gravity (assumed to be 1.09) and RBC volume (assumed to be 3% of body weight). Urine and fecal data were fitted as the cumulative, or daily, amounts (µg or µg/d) excreted over the collection period.

### Kinetic Modeling

Data consisting of tracer and total Se in plasma, RBC, urine, and feces were fitted by a compartmental model (described below) using the WinSAAM software. We assumed that participants were in steady state with respect to Se metabolism for both PK1 and PK2. The model was fitted to PK2 data by changing parameter values while retaining the underlying structure from PK1. Fits of the model to the plasma, RBC, urine, and fecal data were assessed graphically. When the model had been fitted to all participants for ^76^Se (from *Sel*) and ^74^Se (from *SeMet*) separately, the following were estimated for each form: transfer coefficient parameters, L(*i,j*) representing the fraction/h of the contents of compartment *j* transferred to compartment *i*, masses of pools of Se in the body, M(*i*), (µg), transport rates between pools, R(*i,j*), (µg/h), delay times, DT (*i*), (h) and turnover times (h) of each pool, calculated as the reciprocal of the sum of all loss pathways from each compartment. The amounts of Sel-exchangeable Se and SeMet-Se consumed were calculated using the model parameters (27), and termed Diet-Sel and Diet SeMet. These values were combined as described (6).

### Model Description

**Figure 1:**
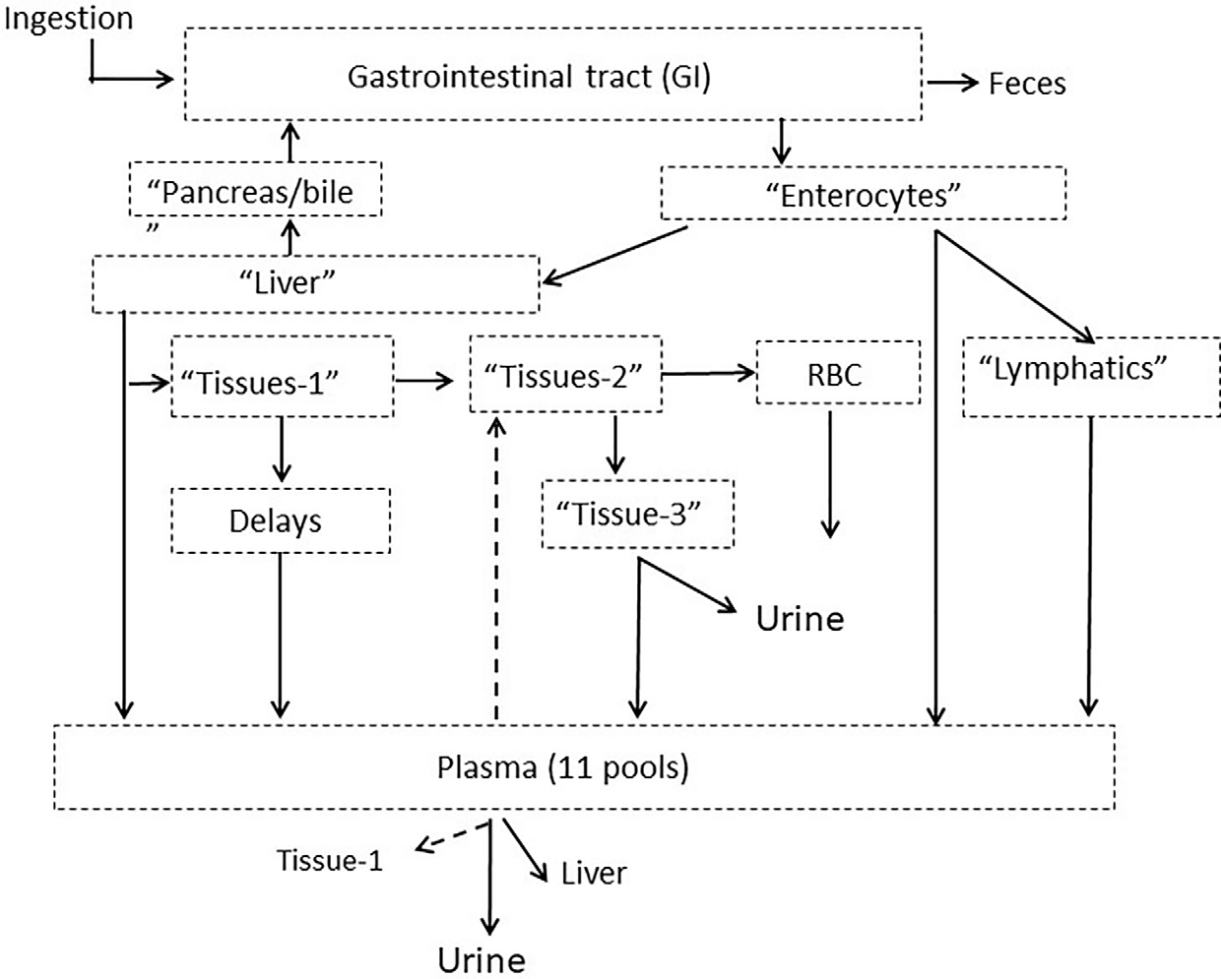
Model schematic for Se metabolism in humans showing compartments grouped into categories with putative physiological labels. Dotted arrows out of plasma indicate pathways that existed for only certain plasma pools. The full model is given in **Supplemental Figure 1**. Published from Wastney et al. (6) by permission of the American Society for Nutrition.

The compartmental model developed in PK1 (6) was used to analyze Se data from PK2. The model consists of Se pools in the gastrointestinal tract, plasma, RBC, liver, and tissues with excretion into urine and feces (**Figure 1**).

The full model is shown in **Supplemental Figure 1**. Following ingestion, a small fraction of tracer (~3%) appeared in plasma (Pl-4) after a 7 h delay and could only be fitted by a portion of the dose entering plasma as a bolus. The rest of the tracer moves through six pools in the gastrointestinal tract (labeled GI-1 to GI-6). Absorption occurs from the first three gastrointestinal pools and Se not absorbed passes through either a 26- or 62-h delay into the colon and then into feces. Absorbed Se taken up by enterocytes, goes either directly to a plasma pool (Pl-1), *via* the lymphatics into a second plasma pool (PL-2, after moving through a chain of compartments) or into liver (Liv-1). After a 2 h delay, Se enters a second liver pool (Liv-2) and is then either released back into the intestinal tract *via* the pancreas or bile (Pancr/bile); travels directly into a plasma pool (Pl-3) or moves into a tissue pool (Tissue-1). Some Se moves from this tissue pool *via* delays ranging from 4- to 175-h into one of six plasma pools (labeled Pl-5 to Pl-10). The plasma pools were differentiated by their turnover rates. From the first tissue pool, some Se also moves into a second tissue pool (Tissue-2) and then either moves into RBC (RBC-1) after a 1,908 h delay, or into a third tissue pool (Tissue-3) and is excreted into urine. For *SeMet* only, exchange occurs between RBC-1 and a second RBC pool (RBC-2) and some Se moves from the third tissue pool into a plasma pool (Pl-11). Some of the plasma pools recycle Se back to the tissues (Pl-3 into Tissue-1, and Pl-9 into Tissue-2); all plasma pools except Pl-11 recycle Se to the liver; and all plasma pools lose Se *via* the kidneys and into urine.

### Statistical Analyses

Results from supplementation were evaluated for changes relative to form and gender and are reported as mean ± SEM. Differences between PK1 and PK2 were calculated as 100 × (PK2 value − PK1 value)/PK1 value. Differences between forms are calculated as [100 × (SeMet − Sel)/Sel] and differences between gender are calculated as [100 × (Female value − Male value)/Male value. Within-subject differences (between *Sel* and *SeMet* and between PK1 and PK2) were tested using two-sided paired t-tests while differences between gender were tested using (unpaired) t-tests, and were considered significant for p < 0.05, as in (23). Statistical software used for the analyses was SAS version 9.1.3 (SAS Institute Inc., Cary, NC, USA). For all participants together, as well as by gender, we report on PK1 *versus* PK2 for *SeMet* and *Sel* and for *SeMet versus Sel* within PK2.

## Results

### Participants and Kinetic Data

Participants (*n* = 20, 7 males, 13 females) were ages 40 ± 3, 39 ± 6, and 40 ± 6 y and weighed 70 ± 3, 77 ± 7, and 66 ± 3 kg for all, males, and females, respectively (**Table 1**). Additional participants are not reported for the following reasons: five males and one female for data discrepancies (primarily lack of fecal samples submitted for analysis), one male who completed PK1 only, one male excluded due to Hashimoto’s disease, and three participants (two males) whose samples were not chemically analyzed but were kept for further analyses (e.g., speciation), but the techniques did not develop before the samples were considered compromised and therefore not retained. Baseline plasma Se concentrations (**Table 1**) were above average US values reported in NHANESIII of 125 ng/ml for men and 122 ng/ml for women (28) indicating that the participants were Se-replete (29). After 2 y of supplementation with 200 µg SeMet/d, plasma Se concentration (i.e., all forms of Se) doubled while RBC Se concentration tripled (**Table 1**). Urinary excretion also nearly tripled, while fecal excretion increased by only 25%. None of the increases in plasma, RBCs, urine, or feces were significantly different in males *vs.* females.

**Table 1:** Age, weight, and Se measurements of participants^1^.

	All, *n = 20*		Males, *n* = 7		Females, *n* = 13	
	PK1	PK2	PK1	PK2	PK1	PK2
Age, *y*	40 ± 3		39 ± 6		40 ± 6	
Weight,^2^ *kg*	70 ± 3		77 ± 7		66 ± 3	
Plasma Se,^3^ *µg/L*	134 ± 3	266 ± 11^*^	141 ± 6	263 ± 16^*^	131 ± 4	267 ± 14^*^
RBC Se, *µg/L*	231 ± 7	649 ± 27^*^	236 ± 13	642 ± 47^*^	227 ± 8	652 ± 35^*^
Urine Se, *µg/d*	71 ± 4	193 ± 6^*^	84 ± 9	209 ± 9^*^	64 ± 4	184 ± 8^*^
Fecal Se, *µg/d*	36 ± 2	45 ± 3^*^	44 ± 4	46 ± 3	31 ± 2	44 ± 4^*^

^1^Values are means ± SEM. ^*^Different from PK1, P < 0.01. For females vs. males, PK2, no values were significant.

^2^Weight not measured at beginning of PK2.

^3^To convert µg to µmol, multiply by 0.0127.

**Figure 2:**
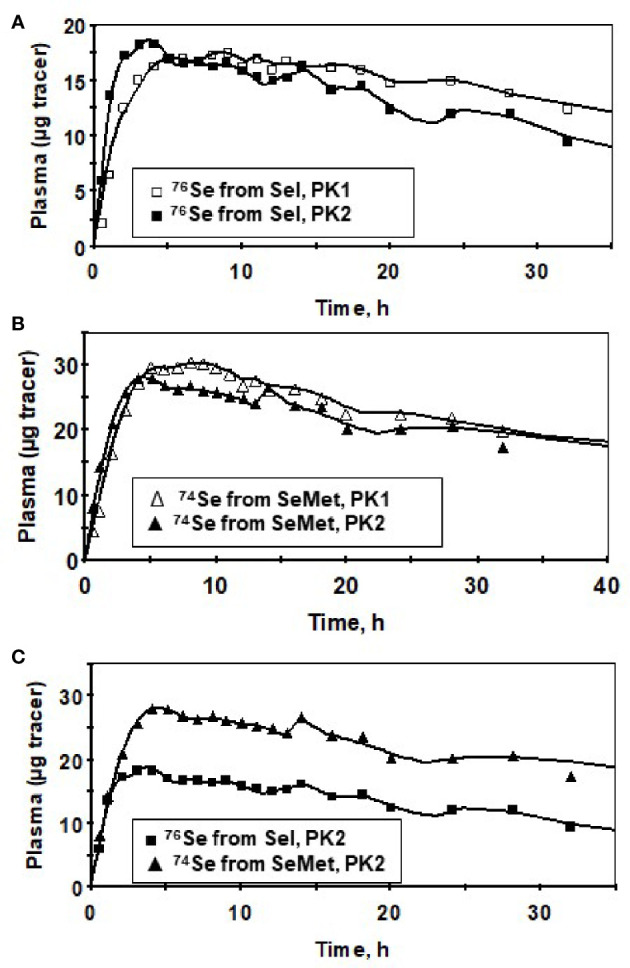
Tracer data for a male subject for *Sel* (^76^Se-*Sel*) before (PK1, ▯), and after (PK2, ◼) Se supplementation **(A)**, *SeMet* (^74^Se -*SeMet*) in plasma for PK1 (∆) and PK2 (▲) **(B)** and for *SeMet* (▲) versus Sel (◼) for PK2 showing similar timing of the peaks between the forms **(C)** for the first 40 h after isotope administration. Symbols are observed values; lines are model-calculated values (**Supplemental Figure 1**).

**Figure 3:**
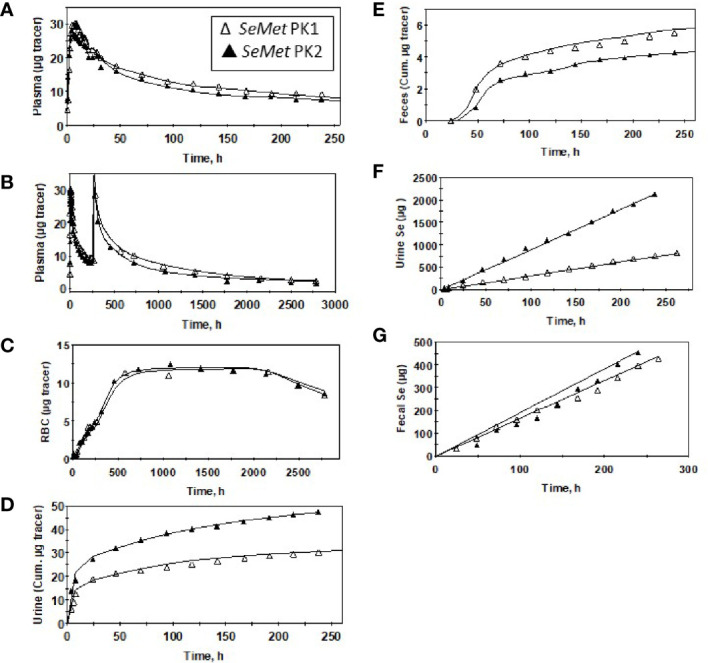
Observed data (∆,▲) and model-calculated (**Supplemental Figure 1**) values (solid lines) for a male subject before (PK1, ∆) and after (PK2, ▲) Se supplementation for *SeMet* (from ^74^Se -SeMet) in plasma for 260 h **(A)** or 2,880 h **(B)** after tracer administration, RBC **(C)**, urine **(D)**, and feces **(E)**; and total Se excreted in urine **(F)** and feces **(G)**.

**Figure 4:**
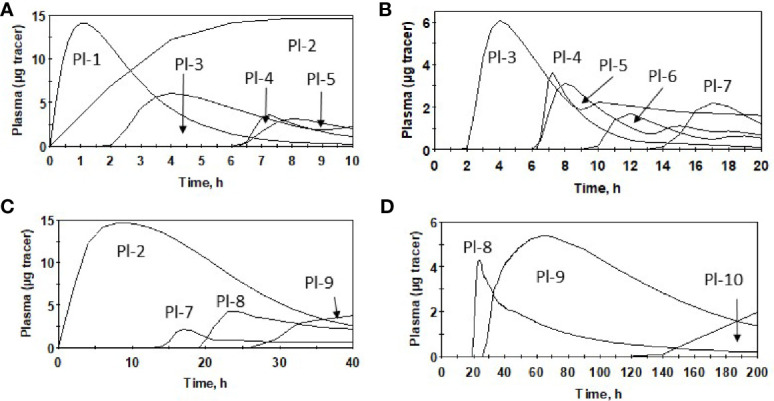
Appearance of oral dose of *SeMet* in plasma pools for males after Se supplementation (PK2), using average parameter values over 0–10 h **(A)**, 0–20 h **(B)**, 0–40 h **(C)** and 0–200 h **(D)** and model (**Supplemental Figure 1**).

Plasma kinetic curves were similar between PK2 and PK1 for *Sel* (**Figure 2A**) and for *SeMet* (**Figure 2B**). However, as in PK1, the curves for the two forms (*SeMet* vs. *Sel*) differed in PK2 (**Figure 2C**) (6). Tracer curves for *SeMet* in PK2 compared with PK1 were slightly lower in plasma and RBC (**Figures 3A–C** **)**, higher in urine (**Figure 3D**), and lower in feces (**Figure 3E**). Cumulative excretion curves of natural Se followed those of the tracers: higher in PK2 than PK1 for urine, but similar for feces (**Figures 3F–G**). Plots of *Sel* and *SeMet* in PK1 *versus* PK2 and *Sel versus SeMet* in PK1 and PK2 for one male subject are provided in **Supplemental Figures 2–5**. (A plot of *Sel versus SeMet* for PK1 for a female is shown in the previous paper (6)). The appearance times of the pools in plasma in PK1 are given in (6) and for PK2 in **Figure 4**.

### Effect of Supplementation on Absorption

With the supplement, Se intake in PK2, estimated as the sum of urinary and fecal excretion, was more than double that in PK1 (**Table 2**). While the percent absorption of both forms remained the same in PK2 as in PK1, because of the increase in SeMet consumed, the Se absorbed more than doubled, from 79 to 202 µg Se/d. As expected, the calculated increase in Se absorbed in PK2 was predominantly from *SeMet.* There were no gender differences in any aspect of absorption in PK2.

**Table 2:** Calculated values for Se intake; absorption of Se, *Sel*, and *SeMet*; daily intake of Se, Sel, SeMet, and amount of Se in humans before (PK1) and after (PK2) supplementation with SeMet^1^.

	All, *n* = 20		Males, n = 7	Females, n = 13
	PK1	PK2	PK2	PK2
Se intake,^2^ *µg/d*	107 ± 6	237 ± 8^**^	255 ± 11^**^	228 ± 11^**^
Se absorption, *%*	73 ± 1	85 ± 0.8^**^	86 ± 1^*^	85 ± 1^**^
Se absorbed, *µg/d*	79 ± 5	201 ± 7^**^	220 ± 10^**^	191 ± 8^**^
Sel : SeMet intake^3^	60:40	36:64	34:66	37:63
*Sel* intake, *µg/d*	64 ± 5	85 ± 11^*^	88 ± 26	84 ± 11
*Sel* absorption, *%*	57 ± 2	58 ± 3	56 ± 5	59 ± 3
*Sel* absorbed,^4^ *µg/d*	36	50	49	50
*SeMet* intake, *µg/d*	43 ± 5	152 ± 11^**^	167 ± 22^**^	144 ± 13^**^
*SeMet* absorption, *%*	97 ± 0.2	97 ± 0.2	98 ± 0.3	97 ± 0.3
*SeMet* absorbed, *µg/d*	42	148	163	140
Total body Se, *mg*	21 ± 1	38 ± 4^**^	42 ± 5^*^	35 ± 5^*^

^1^Values are means ± SEM of those calculated for each participant by the model (**Figure 1**).

*Different from PK1, P < 0.05. ** Different from PK1, P < 0.001. There were no significant differences between males and females.

^2^To convert g to mol, multiply by 0.0127.

^3^Ratio in intake of Sel-exchangeable-Se : SeMet-Se.

^4^Calculated as % absorption × SeMet (or Sel) intake.

### Parameter Changes in PK2 versus PK1 and Between Forms in PK2

The turnover times of the pools in PK2 were like PK1 but the turnover of the largest Se pool (Tissue-3) was faster in PK2 than PK1 (7,308 *versus* 10,325 h, **Supplemental Table 1**). Significant parameter changes in PK2 *vs.* PK1 are listed in **Supplemental Table 2** and included on **Supplemental Figure 6** (for *Sel*) and **Supplemental Figure 7** (for *SeMet*) as dotted arrows; values are given for all participants, males, and females. The largest change for both forms in PK2 *versus* PK1 were increases in urinary excretion pathways (**Supplemental Table 2**).

Differences between forms in PK2 (**Supplemental Table 3**) were that RBC uptake of *SeMet* was 247% higher than for *Sel* and *SeMet* excretion from five of the plasma pools was lower than *Sel.*

### Effect of Supplementation on Pool Sizes, Recycling, and Excretion

The amount of Se in the pools was calculated by assuming that the ratio of SeMet to Sel in body pools was the same as that calculated for the diet. Total Se in the body was estimated to be 80% higher with supplementation (**Table 2**) and all extravascular pools except for the 9-h delay, increased during supplementation (**Supplemental Table 4**).

The plasma pools of Se, expressed as concentrations (using an average bodyweight of 70 kg and plasma volume of 4%, or 2.8 L) also increased about two-fold, except for two pools, Pl-5 and Pl-6 (**Table 3**). Some increases were significant in females but not males (Pl-1, Pl-8, Pl-9, and Pl-10, **Supplemental Table 5**). One plasma pool that was smaller in females in PK1 (Pl-1) (6), increased in PK2 in females but not males, and another pool increased in males but not females (Pl-4) (**Supplemental Table 5**). The distribution of Se in plasma did not change during PK2 vs PK1 (**Table 3** and **Supplemental Table 5**). Recycling to liver increased from Pl-3 for *Sel* and from two pools (Pl-5 and Pl-7) for SeMet (**Table 3** and **Supplemental Table 2**).

**Table 3:** Concentration and distribution of Se in plasma pools before (PK1) and after (PK2) supplementation with SeMet, description of the metabolism of each pool, and putative identification of the pools.

Plasma pool	Plasma “Diet” concentration (µg/L)^1^		Plasma distribution (%)		Description	Putative identification^2^
	PK1	PK2	PK1	PK2		
1	0.73 ± 0.13	1.47 ± 0.19^**^	0.5	0.5	Does not pass through liver. Pool size in females 50% of males in PK1	SeMet
2	4.5 ± 0.5	10.2 ± 1.0^**^	2.7	3.8	Pool size in females 50% of males in PK1. Excretion in urine increased for *SeMet*	Apo B/lipoproteins—from lymphatics
3	2.87 ± 0.62	6.12 ± 1.35^**^	2.2	2.3	Recycling increased for *Sel*	Selenosugar1
4	0.16 ± 0.05	0.40 ± 0.11^*^	0.1	0.1	Increased more in males than females in PK2. Excretion in urine decreased for *SeMet*	
5	1.33 ± 0.35	1.87 ± 0.44	1.0	0.7	Recycling increased for *SeMet*	
6	1.06 ± 0.26	1.04 ± 0.23	0.8	0.4		
7	1.28 ± 0.17	2.00 ± 0.31^*^	1.0	0.7	Recycling increased for *SeMet*	Selenosugar 3
8	4.00 ± 0.52	7.54 ± 0.97^**^	3.0	2.8	Doubles in females in PK2. Excretion in urine increased for *Sel*	
9	15.8 ± 1.5	29.9 ± 5.3^*^	11.9	11.1	Increased 71% in females in PK2. Excretion in urine increased for *SeMet*	GPx3
10	30.3 ± 3.5	69.6 ± 6.5^**^	22.8	25.9	Doubles in females in PK2. Excretion in urine increased for *Sel*	SeP
11	72.0 ± 4.6	138.5 ± 8.1^**^	54.1	51.5	Turnover time is 30 d	Non-specifically incorporated Se (by definition)-albumin
TOTAL	134.0	269.0	100.0	100.0		

*Increased in PK2 vs. PK1, P < 0.05 or **P < 0.01

^1^Values are calculated by the model (**Supplementary Figure 1**). To convert µg to µmol multiply by 0.0127.

^2^Some pools were identified in Wastney et al. (6).

**Figure 5:**
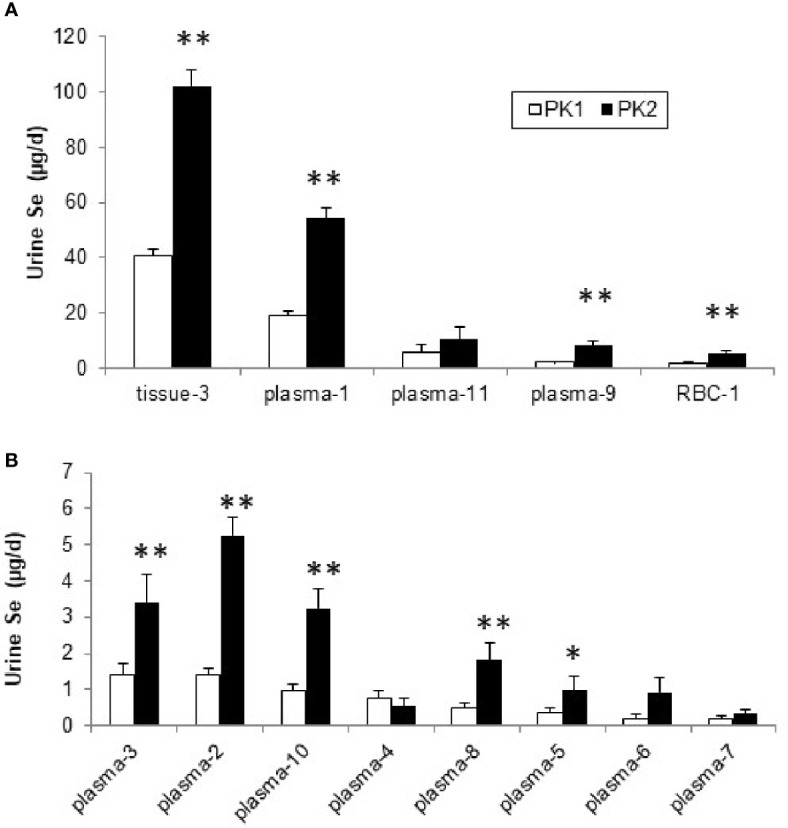
Se (µg/d) excreted into urine from plasma, RBC, and tissue pools before (PK1) and after Se supplementation (PK2) estimated using the model in **Supplemental Figure 1**, for pools with higher excretion rates **(A)** and those with lower rates (<10 µg/d), **(B)**. *Differences are significant for PK2 *vs.* PK1 (P < 0.05), or **(P < 0.01).

The rates of Se urinary excretion from most pools was greater in PK2 than in PK1 but the rates did not change from four plasma pools, (Pl-4, Pl-6, Pl-7, and Pl-11, **Figure 5**). The relative contributions of each pool, expressed as % of total excreted, remained similar in PK1 and PK2 (**Supplemental Table 6**). There were no differences between genders in PK2, however differences were noted in Se form; for *SeMet* excretion increased from plasma Pl-2 and Pl-9 and decreased from Pl-4, and for *Sel* excretion increased from Pl-8 and Pl-10 (**Supplemental Table 2** and **Table 3**).

## Discussion

By comparing Se kinetics before and after 2 yr supplementation with SeMet, we have shown that fractional absorption of SeMet or Sel do not change compared to baseline and, therefore, the amount of Se absorbed increases with supplementation. The amount of Se excreted in urine also increases but does not account for the additional Se absorbed. As a result, there is a net accumulation of Se from SeMet into various body pools. During supplementation, as during baseline *SeMet* is absorbed at a higher rate than *Sel*, and the kinetics of each form are for the most part similar during supplementation to the kinetics during baseline. Only subtle differences were detected between Se forms and these related to the plasma pools contributing Se to urine and recycling to tissues. Some differences were observed between sexes in terms of the size of plasma pools in response to supplementation, the source of Se excreted in urine, and plasma pools that recycle Se to tissues.

### Supplementation Amount and Duration

An assumption of our modeling was that a new steady state had been achieved after 2 y of supplementation. This assumption is supported by our own observations as well as those of others. Burk et al. (29) reported that plasma Se levels approached a plateau in Se-replete participants after 4 mo of supplementation with either 158, 338, and 507 µg/d Se as SeMet. Combs et al. (30) reported plasma concentrations plateauing between 9–12 mo. Se concentration increased in whole blood in Danish participants after daily supplementation for 3 mo with the same dose as in our study (200 µg of Se as SeMet) and were still elevated 4 mo after the end of supplementation (31). In the present study, after 3 mo on 200 µg/d, plasma values were within 13% of levels subsequently reached at 9 mo (23).

### Metabolism by Form

Se form is probably the most influential factor in determining response to supplementation. In agreement with our (5, 25) and others’ studies (19), we found *SeMet* to be almost completely absorbed (97%) while *Sel* absorption was lower (57%); these fractions were the same before and during supplementation. The absolute amount of Se absorbed during supplementation increased, however, as Se intake changed from mainly Sel-like forms to the more readily absorbed SeMet form. The predicted increase in Se intake during supplementation (130 µg/d) was less than the 200 µg/d given as a supplement. The lower-than-expected increase may have been due to reduced dietary Se intake and/or poor compliance with pill-taking or with excreta collection (27). Losses may have been underestimated due to additional loss of Se in breath or desquamation (32).

Kokarnig et al. (33) administered Se in various forms to volunteers and measured non-protein bound Se, or small Se species, in plasma and urine over several hours. At baseline, only Se from Sel could be measured in plasma, but after a single dose of 200 µg Se/d as SeMet, plasma Se increased from <0.1 to 1.2 µg/L at 1 h, and selenosugar increased from <0.1 to 0.63 µg/L at 3 h. Because the stable isotope tracer dose we administered in PK1 was a similar amount (150 µg SeMet), the timing of the appearance tracer in the pools suggests their identity. i.e., Plasma-1 may be SeMet and Plasma-3 may be selenosugar 1 [See **Figure 2** (6)]. The predicted concentration of these compounds are higher in our study (0.7 µg/L for SeMet and 2.9 µg/L for selenosugar), but the plasma Se concentration was 40% higher in our study [134 µg/L *vs.* 95 µg/L in (33)]. Kokarnig et al. (33) reported measuring traces of trimethyselenoselonium ion (TMSe) and methylselenocysteine (MeSeCys) in plasma. We identified at least three other pools in plasma at concentrations <4 µg/L but are not able, without further biochemical analyses, to identify them. We postulated previously that Pool-9 and Pool-10 could be GPX3 and SeP (6). Based on analyses by Combs et al. (34) in adults with similar plasma Se concentrations as the current study, 20% of plasma Se was in GPx3 and 34% in SeP, which agrees with our predictions of the identity of these pools (Pl-9 has 16% of plasma Se, and Pl-10, 30%).

Urinary excretion of Se increased during supplementation, confirming that the kidneys play a major role in the homeostatic regulation of Se (35). In a high-dose human Se-supplementation study conducted by Burk et al. (29), 60% of the doses given were excreted in urine. In the present study, urinary excretion comprised a similar percentage of the supplement. Others report that after ingestion of a single dose (1 mg) of Se as Sel or SeMet, 80% was excreted over 48 h (36). In basal urine, 30–70% of Se in urine could be identified, as selenosugars (36) while others (33) report identifying only 10–20% of the Se species in background urine. However, following ingestion of 200 µg Se as SeMet up to 92% of the species were identified (33), with more than 80% of the Se excreted in urine was as selenosugar 1, about 4% as SeMet, 12% as selenosugar 3, and a trace of TMSe. Following ingestion of 200 µg Se as Sel, selenosugar 1 also accounted for most of the Se (33).

From the kinetics following 2 yr supplementation, we showed that contributions by four of the 11 plasma pools to urine did not increase. We found differences between the forms with respect to which plasma pools contributed to the changes. During supplementation for Se originating from Sel, urinary losses occurred from plasma pools Pl-8 and Pl-10 while for Se from SeMet, changes in urinary losses occurred from three other pools (Pl-2, Pl-4, and Pl-9). Biochemical identification of the plasma pools is required to explain these differences in metabolism. Both forms showed increased urinary excretion from the tissue pool that contained the bulk of Se in the body, but only *Sel* showed increased loss from RBC. The lower absorption of *Sel* was considered by Burk et al. to explain in part the lack of response of plasma selenoproteins to Sel supplementation compared with SeMet (29). Fractional uptake of Se by RBC did not change during supplementation, but Se mass in RBC increased due to the higher rate of uptake. When metabolism of the two forms of Se was compared during supplementation, RBC uptake for *SeMet* was 2.5 times greater than for *Sel*, in agreement with other studies (37).

### Metabolism by Gender

Combs et al. (30) reported a larger increase in urinary Se excretion with Se supplementation in women than in men. We did not find a gender difference in our smaller population, although we found gender differences in response to supplementation of the pools contributing to urine Se: males showed a five-fold increase in urine excretion of *SeMet* from the plasma pool considered to be associated with lipoproteins while females had an almost four-fold increase in loss from the third largest plasma pool. We noted an additional gender difference during supplementation in that the smallest plasma pool, where the bolus entered directly, increased more in males than females. Burk et al. (29) reported a higher level of SeP in males compared to females at baseline, but did not report any gender differences after supplementation (if Plasma Pool-10 is SeP, we also report no gender difference after supplementation). We found that recycling of Se from Sel was increased with supplementation in a gender-specific way; recycling from one plasma pool increased ~2-fold in males but did not change in females, while recycling from another pool increased by 80% in females but did not change in males.

A strength of the present study was that it enrolled healthy non-smokers, examined two tracer forms before and after supplementation, and was able to separate changes in Se metabolism by form and gender and ascribe differences to specific pools and pathways. Previous studies showed increases in plasma and RBC Se concentrations and in urinary and fecal Se excretion during Se-supplementation (18, 29) but were affected by Se form, supplement amount, duration of supplementation, and subject gender and lifestyle characteristics, such as smoking (38). Together these factors limited both the direct comparison and interpretation of studies of Se supplementation as well as the identification of biomarkers of Se status (38). The repeated measures design of this study in which each participant was compared to themselves (i.e., before and following supplementation) eliminated between-subject variance. A repeated-measures design lacks a control group, so effects due to secular trends, such as change in dietary intake, or age-related effects, cannot be evaluated. However, both kinetic studies were conducted in groups of four to five participants over the course of 16 mo, thereby reducing or eliminating seasonality as a potential confounder. A limitation of the current study was the number of males studied compared to females and that speciation was not performed.

### Future Studies

Future studies would identify biochemically the purported plasma pools. Through kinetics we have identified the labeling patterns of these pools, i.e., the time after dosing that each plasma pool has the maximum amount of tracer. By sampling the pools at those times, the identification of the contents of the pools could be determined by speciation analyses. The current studies were in Se-replete participants. Future studies could examine changes in participants with low Se-status. Results from the NPC trial showed that Se supplementation was associated with a significant reduction in total cancer incidence among former smokers (39). This observation suggests that kinetic studies in former smokers may show which pools and pathways of Se metabolism are altered in this population, when compared to our study of non-smokers.

In conclusion, the present study found that supplementation with SeMet increased recycling of Se from specific plasma pools to tissues, and changes in the amount and source of Se excreted in urine. Some changes were Se form- and gender-specific. As expected, the Se mass of most pools increased with SeMet supplementation, because SeMet is incorporated into proteins in place of methionine. Some plasma pools did not increase in mass and these may represent proteins with few methionine residues, or selenoproteins that are regulated, and these proteins may be important in metabolism as they would be expected to respond to supplementation when Se intake is low. All selenoproteins are expected to be identified through the expanding field of selenoproteomics (40).

## Data Availability Statement

The datasets analyzed for this study can be found in the Cancer Data Access System of the Division of Cancer Prevention, National Cancer Institute at https://cdas.cancer.gov/publications/1138/.

## Ethics Statement

The studies involving human participants were reviewed and approved by the NCI Special Studies Institutional Review Board, the Cornell University Committee on Human Subjects, and the Johns Hopkins University Human Subjects Committee (for the BHNRC). The patients/participants provided their written informed consent to participate in this study.

## Author Contributions

BP, GC, and PT designed research. GC conducted research. KP supervised sample analysis. JM, BP, and MW analyzed data. BP and MW wrote the paper. MW had primary responsibility for final content. All authors contributed to the article and approved the submitted version.

## Funding

Supported by Interagency Agreement Y1-SC-0023 between the National Cancer Institute and USDA. This research was supported in part by the Intramural Research Program of the National Institutes of Health, the National Cancer Institute, and the Division of Cancer Epidemiology and Genetics.

## Conflict of Interest

JM was employed by the company Information Management Services, Inc., and MW was employed by Metabolic Modeling Services.

The remaining authors declare that the research was conducted in the absence of any commercial or financial relationships that could be construed as a potential conflict of interest.

## Abbreviations

C, compartment; DT, compartment with a delay time; GPX, glutathione peroxidase; MeSeCys, methylselenocysteine; NPC, Nutritional Prevention of Cancer; PK1, pharmacokinetic study 1 or baseline study; PK2, pharmacokinetic study 2 or supplemented study; Se, selenium; Sel, selenite; *Sel*, ^76^Se from Sel; SeMet, selenomethionine; SeMet, *SeMet*, *^74^*Se from SeMet; SeP, selenoprotein P; SeCys, selenocysteine; SELECT, Selenium and Cancer Prevention Trial; TMSe, trimethyselenoselonium ion.
