# Supplementary material for: Selenium Kinetics in Humans Change Following 2 Years of Supplementation With Selenomethionine
Source: Front Endocrinol (Lausanne). 2021 Mar 29;12:621687. doi: 10.3389/fendo.2021.621687 (PMC8043082; doi:10.3389/fendo.2021.621687)
Supplement: Supplementary file 1 [file DataSheet_1.docx]

**Supplemental Table 1** Turnover times or delay times of Se pools in humans calculated by the model^1^

|  | Turnover or delay time | |  |  | Turnover or delay time | |
| --- | --- | --- | --- | --- | --- | --- |
| Pool | PK1 | PK2 |  | Pool | PK1 | PK2 |
|  | *h* | |  |  | *h* | |
| Colon | 0.1 | 0.1 |  | Pancreas/bile | 37 | 34 |
| Enterocyte | 0.03 | 0.03 |  | Plasma-1 | 0.6 | 0.7 |
| Delay-4 | 4.4 | 4.8 |  | Plasma-2^2^ | 4.6 | 4.1 |
| Delay-7 | 7.4 | 6.7 |  | Plasma-2 | 4.6 | 4.1 |
| Delay-9 | 8.7 | 8.7 |  | Plasma-2 | 4.6 | 4.1 |
| Delay-13 | 12.7 | 13.1 |  | Plasma-2 | 4.6 | 4.1 |
| Delay-19 | 18.8 | 18.2 |  | Plasma-2 | 5 | 4 |
| Delay-30 | 30.2 | 27.4 |  | Plasma-3 | 1.4 | 0.9 |
| Delay-175 | 175 | 142 |  | Plasma-4 | 1.2 | 1.9 |
| GI-1 | 1.3 | 1.3 |  | Plasma-5 | 0.9 | 0.5 |
| GI-2 | 1.3 | 1.3 |  | Plasma-6 | 0.7 | 0.5 |
| GI-3 | 1.3 | 1.3 |  | Plasma-7 | 2.5 | 1.3 |
| GI-4 | 2.5 | 2.9 |  | Plasma-8 | 7.0 | 6.0 |
| GI-5 | 2.5 | 2.9 |  | Plasma-9 | 86 | 43 |
| GI-6 | 1.4 | 2.3 |  | Plasma-10 | 674 | 926 |
| GI-delay-1 | 23 | 30 |  | Plasma-11 | 706 | 666 |
| GI-delay-2 | 87 | 95 |  | RBC-1 | 342 | 805 |
| Kidney | 0.8 | 1.5 |  | RBC-2 | 4796 | 5089 |
| Liver-1 | 0.1 | 0.1 |  | RBC-delay | 1908 | 2031 |
| Liver-2 | 0.2 | 0.2 |  | Tissue-1 | 0.07 | 0.09 |
| Liver-delay | 2.1 | 2.0 |  | Tissue-2 | 57 | 78 |
| Lymphatics | 0.5 | 0.8 |  | Tissue-3 | 10,325 | 7,308 |

^1^Pools are listed alphabetically and refer to the model in Supplemental Fig. 1.

^2^Plasma-2 consists of a chain of five pools with the same turnover times

| **Supplemental Table 2** Comparison of parameter values before (PK1) and after (PK2) Se supplementation in all participants and in each gender, for each Se form^1^ | | | | | | |  |
| --- | --- | --- | --- | --- | --- | --- | --- |
|  | PK2 vs. PK1 | | | | | | |
|  | *Sel* | | |  | *Se-Met* | | |
| Parameter | All *n*=20 | Males *n*=7 | Female *n*=13 |  | All *n*=20 | Males *n*=7 | Female *n*=13 |
|  | *%* | | | | | | |
| Enterocytes from GI-1 | 44 |  |  |  |  |  |  |
| Delay in RBC |  |  |  |  | 6 |  |  |
| Delay-9 | -15 |  |  |  |  |  |  |
| Delay-13 |  |  | -13 |  |  |  |  |
| Pancreas/bile from Liver-1 |  |  |  |  | -23 |  |  |
| Lymphatics from Enterocytes | -12 |  |  |  |  | -21 |  |
| Plasma-2 from lymphatics | -18 |  |  |  | -33 |  | -23 |
| Liver-1 from plasma-6 |  |  | 79 |  |  |  |  |
| Liver-1 from plasma-3 | 84 |  | 152 |  |  |  |  |
| Liver-1 from plasma-5 |  | 93 |  |  | 79 |  | 111 |
| Liver-1 from plasma-7 |  |  |  |  | 86 |  |  |
| To Delay-9 from Tissue-1 | -43 |  |  |  |  |  |  |
| Urine from plasma-3 |  |  | 180 |  |  |  |  |
| Urine from plasma-4 |  |  |  |  | -94 |  |  |
| Urine from tissue-3 | 101 |  | 107 |  | 55 |  |  |
| Urine from RBC-1 | 54 |  |  |  |  |  |  |
| Urine from plasma-10 | 150 | 136 | 156 |  |  |  |  |
| Urine from plasma -9 |  |  |  |  | 553 |  | 389 |
| Urine from plasma-8 | 188 |  | 249 |  |  |  |  |
| Urine from plasma-2 |  |  |  |  | 147 | 551 | 104 |
| Urine from kidney |  | -56 |  |  | -44 |  |  |
| ^1^Parameters refer to the model (Supplemental Fig. 1) and differ significantly (P<0.05) for *PK2 vs. PK1* | | | | | | | |

| **Supplemental Table 3** Pathways that differ significantly by form and by gender during Se supplementation^1^ | | | | | | |
| --- | --- | --- | --- | --- | --- | --- |
|  | Within PK2 | | | | | |
|  | *SeMet vs Sel* | | |  | Females vs. Males | |
| Pathway | All *n*=20 | Males *n*=7 | Females *n*=13 |  | *Sel* | *Se-Met* |
|  | *%* | | | | | |
| Enterocyte from GI-1 | 77 | 114 | 60 |  |  |  |
| Enterocyte from GI-2 | 66 |  | 73 |  |  |  |
| Enterocyte from GI-3 | | -36 |  |  |  |  |
| Delay in RBC | 49 | 28 | 65 |  |  |  |
| Delay-4 | 25 |  |  |  |  |  |
| Delay-9 |  |  |  |  | 32 |  |
| Pancreas/bile from liver | 63 | 140 | 43 |  |  |  |
| Lymphatics from Enterocytes | | | 28 |  |  | 26 |
| Tissue-1 from Liver-2 | -39 |  | -46 |  |  |  |
| Tissue-1 from Plasma-3 | | -74 |  |  |  |  |
| Liver from Enterocytes | | |  |  | 58 |  |
| Liver-1 from Plasma-2 | 20 |  |  |  |  |  |
| Plasma-3 from Liver-2 | -23 | -20 | -25 |  |  |  |
| Delay-13 from Tissue-1 | | |  |  | -44 |  |
| Uptake by RBC | 247 |  | 150 |  |  |  |
| Urine from Plasma-3 | -75 |  | -77 |  | 814 |  |
| Urine from Tissue-3 | -68 |  | -69 |  |  |  |
| Urine from RBC-1 | -38 | -54 |  |  |  |  |
| Urine from Plasma-10 | -60 | -64 |  |  |  |  |
| Urine from Plasma-9 | -73 | -80 |  |  |  |  |
| Urine from Plasma-8 | -84 |  | -81 |  |  |  |
| Urine from Plasma-2 | -41 |  |  |  |  |  |
| Urine from Kidney | 98 | 533 |  |  |  |  |
| ^1^Parameters refer to the model (Supplemental Fig. 1) and differ significantly (P<0.05) for *Sel* vs. *SeMet* or by gender for each form. | | | | | | |

**Supplemental Table 4 C**alculated mass of Se in pools before (PK1) and after (PK2) Se supplementation expressed as amount for all participants and % change for all participants and each gender^1^

|  | All, n=20 | |  | All, n=20 | Males, n=7 | | Females, n=13 | |
| --- | --- | --- | --- | --- | --- | --- | --- | --- |
| Pool | PK1 | PK2 |  | PK2 vs. PK1 | | | | |
|  | *µg^2^* | |  | *% increase* | | | | |
| Enterocytes | 0.12 | 0.34 |  | *173*** | | *183** | | *166**#* |
| Lymphatics | 0.33 | 1.09 |  | *230*** | | *177** | | *284*** |
| Delay-7 | 0.76 | 1.88 |  | *148*** | | *155* | | *144** |
| Tissue-1 | 0.99 | 2.27 |  | *130*** | | *144** | | *121** |
| Liver-1 | 1.43 | 3.15 |  | *120*** | | *105* | | *138*** |
| Liver-2 | 2.80 | 6.28 |  | *124*** | | *85** | | *178** |
| Kidney | 10.2 | 32.4 |  | *216*** | | *297** | | *168** |
| Delay-4 | 11.5 | 35.1 |  | *204** | | *249* | | *160** |
| Delay-175 | 12.4 | 29.6 |  | *139*** | | *78** | | *186*** |
| Delay-30 | 12.8 | 33.8 |  | *163** | | *110* | | *229*** |
| Delay-13 | 17.8 | 44.4 |  | *149** | | *159* | | *141** |
| Delay-9 | 23.2 | 33.0 |  | *42* | | *0* | | *93* |
| Delay-19 | 27.5 | 52.8 |  | *92** | | *109* | | *78* |
| Liver-delay | 38.2 | 60.3 |  | *58** | | *12* | | *143** |
| Pancreas/bile | 67.1 | 142.0 |  | *112** | | *94* | | *123* |
| RBC-1 | 142 | 359 |  | *153*** | | *180** | | *139*** |
| RBC-delay | 151 | 480 |  | *219*** | | *183*** | | *243*** |
| RBC-2 | 234 | 624 |  | *167*** | | *153*** | | *176*** |
| Plasma^3^ | 365 | 729 |  |  | |  | |  |
| Tissue-2 | 266 | 666 |  | *150*** | | *80** | | *200*** |
| Tissue-3 | 19,434 | 34,821 |  | *79*** | | *68** | | *87** |
| TOTAL | 20,820 | 38,159 |  |  | |  | |  |

^1^Pools refer to the model (Supplemental Fig. 1) and mass was calculated from a diet containing organic and inorganic Se. (See text for details). *Different from PK1, P <0.05. ** Different from PK1, P ≤ 0.001. # Males different from females, PK2, P< 0.05.

^2^To convert from µg to µmol multiply by 0.0127.

^3^See Table 1 for gender differences in total Se in plasma and Supplemental Table 5 for pools within plasma

**Supplemental Table 5** Calculated increase in mass of Se in plasma pools before (PK1) and after (PK2) Se supplementation by gender and the distribution of Se among plasma pools in PK1, and the change in distribution in PK2, for all participants and each gender^1^

|  | Diet Mass^2^ | |  | Distribution within plasma | | | | |
| --- | --- | --- | --- | --- | --- | --- | --- | --- |
|  | PK2 vs. PK1 | |  | PK1 |  | PK2 vs. PK1 | | |
| Pool | Males, n=7 | Females, *n*=13 |  | All, n=20 |  | All, *n*=20 | Males, *n*=7 | Females, n=13 |
|  | *% Increase* | |  | *%* |  | *% Increase* | | |
| Plasma-1 | 56 | 155*^3^ |  | 0.55 |  | 0 | -16 | 21* |
| Plasma-2 | 86* | 172** |  | 3.34 |  | 14 | 1* | 29* |
| Plasma-3 | 174 | 76 |  | 2.14 |  | 6 | 48 | -17 |
| Plasma-4 | 183 | 104^#^ |  | 0.12 |  | 23 | 53 | -3 |
| Plasma-5 | 62 | 27 |  | 0.99 |  | -30 | -13 | -40 |
| Plasma-6 | 9 | -9 |  | 0.79 |  | -51 | -41 | -57 |
| Plasma-7 | 26 | 73 |  | 0.96 |  | -22 | -32 | -18 |
| Plasma-8 | 76* | 96* |  | 2.99 |  | -6 | -5* | -7* |
| Plasma-9 | 120 | 71* |  | 11.78 |  | -6 | 19 | -19* |
| Plasma-10 | 78 | 171** |  | 22.60 |  | 15 | -4 | 29* |
| Plasma-11 | 81* | 99** |  | 53.74 |  | -4 | -2* | -5* |
| TOTAL |  |  |  | 100.0 |  |  |  |  |

^1^Pools refer to the model in Supplemental Fig. 1.

^2^Diet mass is the mass calculated from a diet containing both Sel-exchangeable Se and SeMet. See text for details.

^3^*Different from PK1, P <0.05. ** Different from PK1, P ≤ 0.001. # Different from males, P<0.05.

**Supplemental Table 6**. Source of urinary Se (amount and %) for all participants before (PK1) and after (PK2) Se supplementation and for each gender the % change in source with supplementation^1^

|  | **All, *n*=20** | | | |  | **Males, *n*=7** | **Females, n=13** |
| --- | --- | --- | --- | --- | --- | --- | --- |
| Pool | PK1 | PK2 | PK1 | PK2 |  | PK2 vs. PK1 | |
|  | *µg/d^2^* | | *% from pool^3^* | |  | *% change in source* | |
| Tissue-3 | 40.6±2.4 | 102.2±5.7**^4^ | 54.0 | 51.7 |  | -4** | -5** |
| Plasma-1 | 18.8±2.1 | 54.2±3.9** | 24.9 | 27.4 |  | 11** | 9** |
| Plasma-11 | 6.1±2.4 | 10.7±4.4 | 8.0 | 5.4 |  | -44 | -32 |
| Plasma-9 | 2.1±0.3 | 8.2±1.5** | 2.8 | 4.2 |  | 29 | 59** |
| RBC-1 | 2.0±0.2 | 5.8±0.4** | 2.7 | 2.9 |  | 6** | 11** |
| Plasma-3 | 1.4±0.3 | 3.4±0.8** | 1.9 | 1.7 |  | -25 | -5** |
| Plasma-2 | 1.4±0.2 | 5.3±0.5** | 1.9 | 2.7 |  | 25** | 48** |
| Plasma-10 | 1.0±0.2 | 3.2±0.5** | 1.3 | 1.6 |  | 19 | 31** |
| Plasma-4 | 0.7±0.2 | 0.5±0.2 | 1.0 | 0.3 |  | -313 | -70 |
| Plasma-8 | 0.5±0.1 | 1.8±0.5* | 0.6 | 0.9 |  | 47 | 20* |
| Plasma-5 | 0.4±0.1 | 1.0±0.4* | 0.5 | 0.5 |  | -27 | 6 |
| Plasma-6 | 0.2±0.1 | 0.9±0.4 | 0.3 | 0.5 |  | 29 | 112 |
| Plasma-7 | 0.2±0.1 | 0.3±0.1 | 0.3 | 0.2 |  | 66 | -50 |
| TOTAL | 75.3 | 197.6 | 100 | 100 |  |  |  |

^1^Values are means ± SEM of those calculated for each participant by the model (Supplemental Fig. 1)

^2^To convert from g to mol multiply by 0.0127.

^3^The contribution was calculated as 100 X [diet Se (i.e., sum of Sel-exchangeable Se and Se-Met Se) excreted from each pool per d/total Se excreted in urine per d].

^4^*Different from PK1, P <0.05. ** Different from PK1, P ≤ 0.01. No significant differences in % change from source occurred between genders in PK2.

Supplemental Figure 1. Compartmental model for Se metabolism in humans showing compartment labels. The numbers in the top right of each compartment were those used in the WinSAAM files. Published from Wastney et al. [[1](#_ENREF_1)] by permission of the American Society for Nutrition.

Tissue-1

Plasma

(Pl)

Gastrointestinal tract (GI)

“Liver”,

(Liv)

RBC

“Tissue-1”

“Lymphatics”

“Kidney”

Feces

Urine

7*h*

4*h*

9*h*

13*h*

19*h*

30*h*

175*h*

“Tissue-2”

“Tissue-3”

“Enterocytes”

Ingestion

GI-2

GI-1

GI-5

GI-3

GI-6

GI-4

62 *h*

26 *h*

Colon

Pl-1

Pl-2

Pl-3

Pl-4

Pl-5

Pl-6

Pl-7

Pl-8

Pl-9

Pl-10

Pl-11

Delay

times, *h*

Liv-1

Liv-2

2*h*

Liv-1

1908*h*

RBC-1

RBC-2

*1*

*60,61,62 63,70*

*69*

*38*

*39*

*23*

*24*

*28*

*34*

*66*

*41*

*13*

*33*

*29*

*64*

*37*

*31*

*20*

*6*

*22*

*27*

*21*

*3*

*4*

*5*

*14*

*15*

*17*

*18*

*9*

Urine

“Pancreas/bile”

*19*

*7*

*10*

*25*

*32*

*36*

*65*

*67*

*40*

*35*

Supplemental Figure 2. PK1 Sel vs SeMet: Tracer data for a male subject for *Sel* (^76^Se-Sel, ), and *SeMet* (^74^Se –SeMet, ∆). Symbols are observed values; lines are model-calculated values (Supplemental Figure 1) for plasma 0-40 h after isotope administration (*A*), plasma 0-260 h (*B*), plasma 0-2800 h (*C*), RBC (*D*), urine (*E*), Feces (*F*) and for total Se excreted in urine (*G*) and feces (*H*).

Supplemental Figure 3. PK2 Sel vs SeMet: Tracer data for a male subject for *Sel* (^76^Se-Sel, ) and *SeMet* (^74^Se –SeMet, ▲). Symbols are observed values; lines are model-calculated values (Supplemental Figure 1) for plasma 0-40 h after isotope administration (*A*), plasma 0-260 h (*B*), plasma 0-2800 h (*C*), RBC (*D*), urine (*E*), feces (*F*) and for total Se excreted in urine (*G*) and feces (*H*).

□ *Sel* PK1

*■ Sel* PK2

**A**

**B**

**C**

**G**

**E**

**F**

**D**

**H**

Supplemental Figure 4. *Sel* (^76^Se-Sel) in PK2 (l) vs. PK1 () for a male subject. Symbols are observed values; lines are model-calculated values (Supplemental Figure 1) for plasma 0-40 h after isotope administration (*A*), plasma 0-260 h (*B*), plasma 0-2800 h (*C*), RBC (*D*), urine (*E*), feces (*F*) and for total Se excreted in urine (*G*) and feces (*H*).

Supplemental Figure 5. *SeMet* (^74^Se –SeMet) in PK2 (▲) vs. PK1 (∆) for a male subject. Symbols are observed values; lines are model-calculated values (Supplemental Figure 1) for plasma 0-40 h after isotope administration (*A*), plasma 0-260 h (*B*), plasma 0-2800 h (*C*), RBC (*D*), urine (*E*), and feces (*F*).

Supplemental Figure 6 Model for movement of Se tracer through the body compartments with changes in parameter values during supplementation shown by dotted arrows. The numerical value (%) is listed by pathways (arrows) that change; where one value is shown there was no difference between males and females, where 3 values are shown, the top value is for all participants, the middle value for males and bottom value for females for *Sel* (^76^Se-Sel).

Supplemental Figure 7 Model for movement of Se tracer though the body compartments with changes in parameter values during supplementation shown by dotted arrows. The numerical value (%) is listed by pathways (arrows) that change; where one value is shown there was no difference between males and females, where 3 values are shown, the top value is for all participants, the middle value for males and bottom value for females for *SeMet* (^74^Se -SeMet).

[1] M.E. Wastney, G.F. Combs, Jr., W.K. Canfield, P.R. Taylor, K.Y. Patterson, A.D. Hill, J.E. Moler, and B.H. Patterson, A human model of selenium that integrates metabolism from selenite and selenomethionine. The Journal of nutrition 141 (2011) 708-17.
